# A statistical approach for the production of thermostable and alklophilic alpha-amylase from *Bacillus amyloliquefaciens* KCP2 under solid-state fermentation

**DOI:** 10.1007/s13205-014-0213-1

**Published:** 2014-04-11

**Authors:** Vimal S. Prajapati, Ujjval B. Trivedi, Kamlesh C. Patel

**Affiliations:** BRD School of Biosciences, Sardar Patel University, Sardar Patel Maidan, Vadtal Road, Satellite Campus, Post Box No. 39, Vallabh Vidyanagar, 388-120 Gujarat India

**Keywords:** Thermostable alpha-amylase, Plackett–Burman design, Response surface methodology, Solid-state fermentation, *Bacillus amyloliquefaciens* KCP2

## Abstract

The bacterial strain producing thermostable, alklophilic alpha-amylase was identified as *Bacillus amyloliquefaciens* KCP2 using 16S rDNA gene sequencing data (NCBI Accession No: KF112071). Medium components were optimized through the statistical approach for the synthesis of alpha-amylase by the organism under solid-state fermentation using wheat bran as the substrate. The medium components influencing the enzyme production were identified using a two-level fractional factorial Plackett–Burman design. Among the various variables screened, starch, ammonium sulphate and calcium chloride were found to be most significant medium components. The optimum levels of these significant parameters were determined employing the response surface Central Composite design which significantly increased the enzyme production with the supplementation of starch 0.01 g, ammonium sulphate 0.2 g and 5 mM calcium chloride in the production medium. Temperature and pH stability of the alpha-amylase suggested its wide application in the food and pharmaceutical industries.

## Introduction

Amylases find potential application in number of industrial processes such as bread making, brewing, starch processing, pharmacy, textile industries. Alpha-amylase is an extracellular enzyme that randomly cleaves the 1,4 α-d-glucosidic linkages between adjacent glucose units in the linear amylose chain. It is secreted as primary metabolite of microorganisms and its production is a growth-related process (Kammoun et al. 2008). It possesses approximately 25–33 % share of the world’s marketable enzymes. Microbial alpha-amylases are the most stable and produced more economically compared to plant and animal alpha-amylases (Gupta et al. 2003).

To meet the growing demands of amylase for industrial application, it is necessary to produce the highly efficient enzymes at large scale with reduced production cost (Haq et al. 2003). Submerged fermentation (SmF) has been traditionally used for the production of industrially important enzymes because of the ease of handling and greater control of environmental factors such as temperature and pH. Use of agro-industrial residues as the substrate for the fermentation has growing interests as they are inexpensive energy-rich resources and also eliminates large-scale accumulation of the biomass (Pandey et al. 2000; Ramachandran et al. 2007). Solid-state fermentation has been generally referred useful for agro-residues utilization (Pandey et al. 2000; Babitha et al. 2007; Binod et al. 2007), although most of the commercial processes are based on submerged fermentation. The growth and the enzyme production by the organisms are strongly influenced by medium composition, and hence optimization of the medium components may lead to improved enzyme productivity (Djekrif et al. 2006).

The main strategy used for optimizing the medium composition is by changing one medium component as a parameter and keeping the others at a constant level (Prajapati et al. 2013). Such optimization studies do not consider the interaction effects among the variables which influence the overall process for the production of a desired metabolite (Silva et al. 2001). Single variable optimization methods are not only tedious, but also can lead to misinterpretation of results, especially because the interaction effects between different factors are overlooked (Wenstet-Botz 2000). Limitations of the single factor optimization can be eliminated by employing response surface methodology (RSM) which is used to explain the combined effects of all the factors in a fermentation process (Elibol 2004). Response surface methodology may be summarized as a collection of experimental strategies, mathematical methods and statistical inference for constructing and exploring an approximate functional relationship between a response variable and a set of design variables.

In the present study, Plackett–Burman design is used for identifying various nutrients as significant variables influencing alpha-amylase production by *Bacillus amyloliquefaciens* KCP2. The levels of the significant variables are further optimized using response surface methodology.

## Materials and methods

### Strain isolation and identification

Bacterial strain isolated from municipal food waste samples collected from Vallabh Vidyanagar, Gujarat, India, on Luria agar (LA) was screened for amylase production on starch agar plate. Culture was maintained at 4 °C on Bushnell Hass agar (BHA) slants containing 1 % starch. Bushnell Hass mineral salt solution has the following composition (g/l): MgSO_4_ 0.2, CaCl_2_ 0.02, KH_2_PO_4_ 1.00, K_2_HPO_4_ 1.00, NH_4_NO_3_ 1.00 and FeCl_3_ 0.05 (pH 7.0). Genomic DNA of the bacterial isolate was extracted and the 16S rDNA gene was amplified using the universal primers (F, 5′ AGAGTTTGATCCTGGCTCAG 3′; R, 5′ GGTTACCTTGTTACAGCTT 3′). Amplification was carried out in a thermal cycler (Applied Biosystems) with reaction profile: initial denaturation at 95 °C for 1 min, followed by 30 cycles of denaturation at 95 °C for 30 s, annealing at 55 °C for 45 s, extension at 72 °C for 45 s and finally extension at 72 °C for 5 min. The purified PCR product was sequenced and the phylogenic relationship of the isolate was determined by comparing the sequence data with the existing sequences available through the gene bank database of the National Center for Biotechnology Information (NCBI).

### Preparation of inoculum

A volume of 50 ml of nutrient broth taken in a 250-ml Erlenmeyer flask was inoculated with a loop full of cells from a 24-h-old culture and kept at 37 °C in a rotary shaker. After 18 h of incubation, an appropriate aliquot of inoculum was added to get one optical density unit in all the experimental flasks.

### Enzyme production, extraction and assay procedure

The experiments were performed according to the design matrix (Tables 2, 4) in 250-ml Erlenmeyer flasks containing 5 g wheat bran as solid substrate and 10 ml of the salt solution to provide the adequate moisture content. After inoculation, all the experimental flasks were incubated under static condition at 37 ± 2 °C and were harvested after 72 h interval followed by the enzyme extraction with 40 ml of 0.05 M phosphate buffer (pH 8.0) on a rotary shaker at 150 rpm for 30 min at 25 °C. The content was filtered through muslin cloth, centrifuged at 8,000 rpm for 25 min and the clear supernatant was used for determining alpha-amylase activity, which is expressed as U/gds (Units/gram dry substrate). The reaction mixture consisted of 1.0 ml of 1 % starch, 0.9 ml 0.05 M phosphate buffer (pH 8.0), and 0.1 ml of enzyme extract. After 10 min of incubation at 65 °C, the liberated reducing sugars were estimated by the dinitrosalicylic acid (DNS) method of Miller (1959). The colour developed was read at 560 nm using a spectrophotometer (Shimadzu UV-160). TLC analysis of products of enzymatic hydrolysis of starch indicated that it is alpha-amylase since the major end product was found to be maltose.

### Effect of the pH and temperature on the enzyme activity and stability

Enzyme produced by the *B. amyloliquefaciens* KCP2 was assayed at different temperatures and pH ranging 30–90 °C and 4–10, respectively. Stability of enzyme was tested by incubating in buffers of different pH (4–9) at 30 °C up to 180 min and residual activity was determined after each 10 min of incubation under standard assay condition. The thermal stability was studied by incubating enzymes at various temperatures (30–90 °C) and residual activity was measured after each 10 min of interval up to 180 min under standard assay condition.

### Screening of significant variables using Plackett–Burman design

Plackett–Burman design is a powerful technique for screening and evaluating the important variables that influences the response (Plackett and Burman 1946). This technique significantly decreases the number of experiments needed to decide the important variables. In the present study, various medium components such as dextrose, starch, lactose, soya bean meal, yeast extract, ammonium sulphate, ammonium nitrate and calcium chloride were investigated as variables using PB design to identify the components that significantly affected alpha-amylase production. In PB design each selected variable was considered at two levels, high (+) and low (−) as shown in Table 1. Using the selected levels for each variable and three-dummy variable setup with 12 runs of experiment was generated using the software as shown in Table 2. Each row represents a trial, and each column represents an independent (assigned) or dummy (unassigned) variables. The effect of each variable was determined by the following equation:

**Table 1:** Variables representing medium components used in Plackett–Burman design

Variables	Medium components	Positive values	Negative values
X1	Dextrose	1.5 g	0.05 g
X2	Starch	1.5 g	0.05 g
X3	Lactose	1.5 g	0.05 g
X4	Soy bean meal	1 g	0.05 g
X5	Yeast extract	1 g	0.05 g
X6	Ammonium sulphate	1 g	0.05 g
X7	Ammonium nitrate	1 g	0.05 g
X8	Calcium chloride	5 mM	1 mM

**Table 2:** Design matrix and experimental results of Plackett–Burman design

Run no.	Components											Alpha-amylase production (U/gds)
	X1	X2	X3	X4	X5	X6	X7	X8	D1	D2	D3	
1	1	1	−1	1	1	1	−1	−1	1	1	−1	9.334
2	−1	1	1	−1	1	1	1	−1	−1	−1	1	0.502
3	1	−1	1	1	−1	1	1	1	−1	−1	−1	3.702
4	−1	1	−1	1	1	−1	1	1	−1	−1	−1	12.746
5	−1	−1	1	−1	1	1	−1	1	1	1	−1	16.474
6	−1	−1	−1	1	−1	1	1	−1	1	1	1	9.308
7	1	−1	−1	−1	1	−1	1	1	1	1	1	21.73
8	1	1	−1	−1	−1	1	−1	1	−1	−1	1	10.26
9	1	1	1	−1	−1	−1	1	−1	1	1	−1	5.50
10	−1	1	1	1	−1	−1	−1	1	1	1	1	19.17
11	1	−1	1	1	1	−1	−1	−1	−1	−1	1	27.63
12	−1	−1	−1	−1	−1	−1	−1	−1	−1	−1	−1	39.05

D1–D3 represent dummy variables

1 $$E \, \left( {\text{Xi} } \right) \, = \, 2\left( {\varSigma \, \text{Mi}^{ + } {-} \, \text{Mi}^{-} } \right),$$

where *E* (Xi) is the concentration effect of the tested variable Mi^+^ and Mi^−^ representing the alpha-amylase production from trials where the variable (Xi) measured was presented at high and low concentrations, respectively. *N* is the total number of trials, i.e. 12. Experimental error was estimated by calculating the variance among the dummy variables as:

2 $$V_{\text{eff}} = \, \varSigma \, \left( {E_{d} } \right)^{2} / \, n,$$

where *V*_eff_ is the variance of the effect of level, *E*_*d*_ is the effect of the level for the dummy variables and *n* is the number of dummy variables used in the experiment. The standard error (SE, Es) of concentration effect was the square root of variance of an effect, and the significance level (*P* value) of each concentration effect was determined using the Student’s *t* test.

3 $$t\left( {\text{Xi} } \right) \, = \, E\left( {\text{Xi} } \right) \, / \, \text{Es} ,$$

where *E* (Xi) is the effect of variable Xi.

### Response surface methodology

The concentration of the medium components found as a significant variable and the interaction effects between them which may influence the alpha-amylase production significantly were analysed and optimized by response surface Central Composite design (CCD). RSM is useful for small number of variables (up to five) but is impractical for large number of variables, due to high number of experimental runs required. In the present study, concentrations of the three major medium components, starch, ammonium sulphate and calcium chloride (identified by Plackett–Burman design) were optimized, keeping temperature, pH, moisture and inoculum size constant.

According to the design, the total number of treatment combinations is 2^*k*^ + 2*k* + no, where *k* is the number of independent variables and no is the number of repetition of experiments at the central point. Each factor in the design was studied at five different levels (−α, −1, 0, +1, +α) as shown in Table 3. All variables were set at a central coded value of zero. The minimum and maximum ranges of variables were determined on the basis of our previous experiments. The full experimental plan with respect to their values in actual and coded form is presented in Table 4. The alpha-amylase activity was measured in triplicate for all 20 different experimental runs. The enzyme production was analysed by using a second-order polynomial equation, and the data were fitted into the equation by multiple regression procedure. The model equation for analysis is given as:

**Table 3:** Experimental range and levels of the independent variables of selected components used for response surface Central Composite design

Variables	Components	Range	Levels of variable studied				
			−α	−1	0	+1	+α
X1	Starch (w/w)	0.01–0.50	−0.15	0.01	0.25	0.50	0.66
X2	Ammonium sulphate (w/w)	0.01–0.20	−0.05	0.01	0.10	0.20	0.264
X3	Calcium chloride (mM)	0.50–5.00	−1.03	0.50	2.75	5.00	6.53

**Table 4:** Full experimental Central Composite design with coded and actual level of variables and the response function

Run no.	A: starch w/w		B: ammonium sulphate w/w		C: calcium chloride (mM)		Alpha-amylase (U/gds)	
	Actual level	Coded level	Actual level	Coded level	Actual level	Coded level	Observed	Predicted
1	0.01	−1	0.01	−1	0.5	−1	49.27	48.96
2	0.5	+1	0.01	−1	0.5	−1	46.78	46.04
3	0.01	−1	0.2	+1	0.5	−1	48.55	48.78
4	0.5	+1	0.2	+1	0.5	−1	42.77	41.82
5	0.01	−1	0.01	−1	5	+1	51.08	52.78
6	0.5	+1	0.01	−1	5	+1	46.86	47.39
7	0.01	−1	0.2	+1	5	+1	60.01	61.48
8	0.5	+1	0.2	+1	5	+1	51.21	52.06
9	−0.15	−α	0.105	0	2.75	0	54.88	53.40
10	0.66	+α	0.105	0	2.75	0	42.60	43.02
11	0.255	0	−0.05	−α	2.75	0	46.95	46.60
12	0.255	0	0.26	+α	2.75	0	51.10	50.37
13	0.255	0	0.105	0	−1.03	−α	45.38	46.80
14	0.255	0	0.105	0	6.53	+α	61.10	58.62
15	0.255	0	0.105	0	2.75	0	48.59	47.94
16	0.255	0	0.105	0	2.75	0	47.07	47.94
17	0.255	0	0.105	0	2.75	0	48.93	47.94
18	0.255	0	0.105	0	2.75	0	46.10	47.94
19	0.255	0	0.105	0	2.75	0	48.42	47.94
20	0.255	0	0.105	0	2.75	0	48.34	47.94

4 $$Y \, = \, \beta_{0} + \, \sum \, \beta i\text{Xi} \, + \, \sum \, \beta ii\text{Xi}^{2} + \sum \, \beta ij\text{XiXj} ,$$

where *β*_0_, *βi*, *βii* and *βij* represent the constant process effect in total, the linear, quadratic effect of *Xi* and the interaction effect between Xi and Xj, respectively, for the production of alpha-amylase. Later, an experiment was run using the optimum values for variables given by response optimization to confirm the predicted value and experimental value of enzyme production.

### Software and data analysis

The results of the experimental design were analysed and interpreted using Design Expert Version 8.0 (Stat-Ease Inc., Minneapolis, Minnesota, USA) statistical software.

## Results

### Identification of the bacterial isolate

**Fig. 1:**
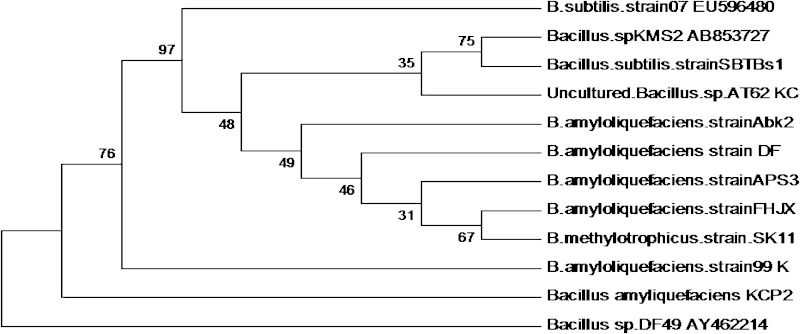
Phylogenetic relationship on the basis of homology index for a bacterial isolate *B. amyloliquefaciens* KCP2

A 800-bp size 16S rDNA sequence of the isolate was obtained through PCR amplification and sequencing. The sequence was subjected to a multiple sequence alignment using the BLAST programme of NCBI. The sequence showed a homology of 99 % with *B. amyloliquefaciens.* The sequence was deposited in the gene bank of the NCBI (Accession No: KF112071). The phylogenetic tree (Fig. 1) was drawn using bioinformatics software MEGA 4.0 (Tamura et al. 2007) after alignment of the sequences with the Clustal X software.

### Activity and stability of alpha-amylase from *B. amyloliquefaciens* KCP2

**Fig. 2:**
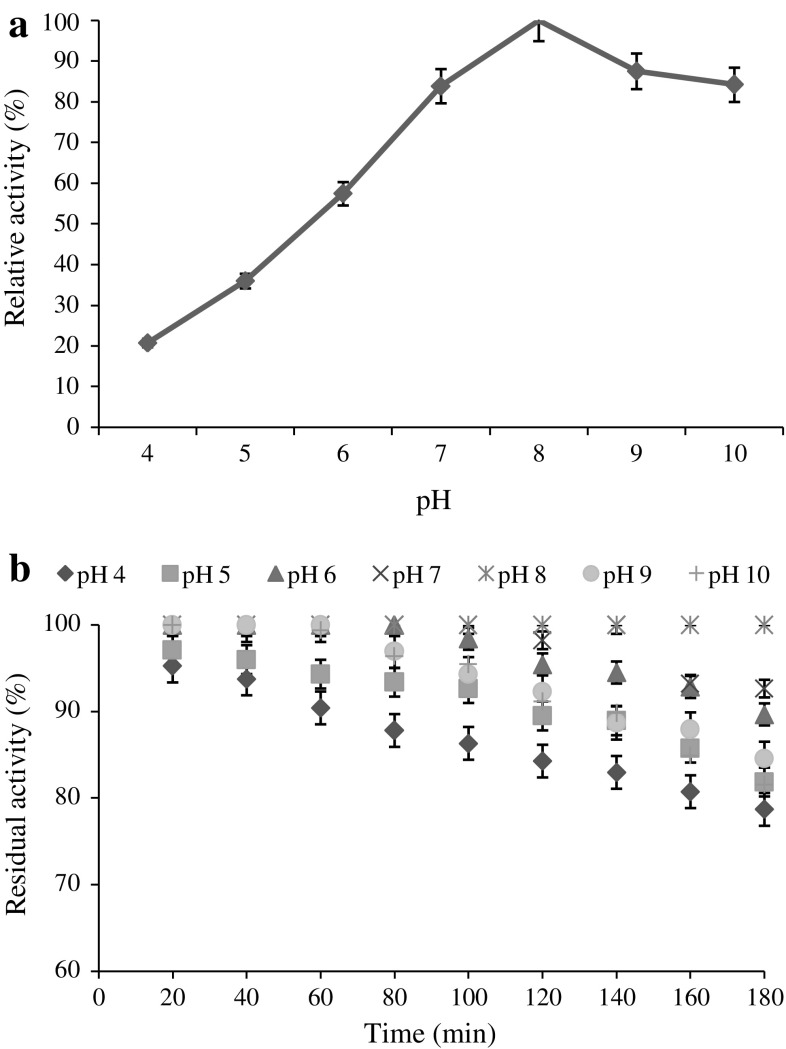
Effect pH on enzyme activity (**a**) and stability (**b**)

**Fig. 3:**
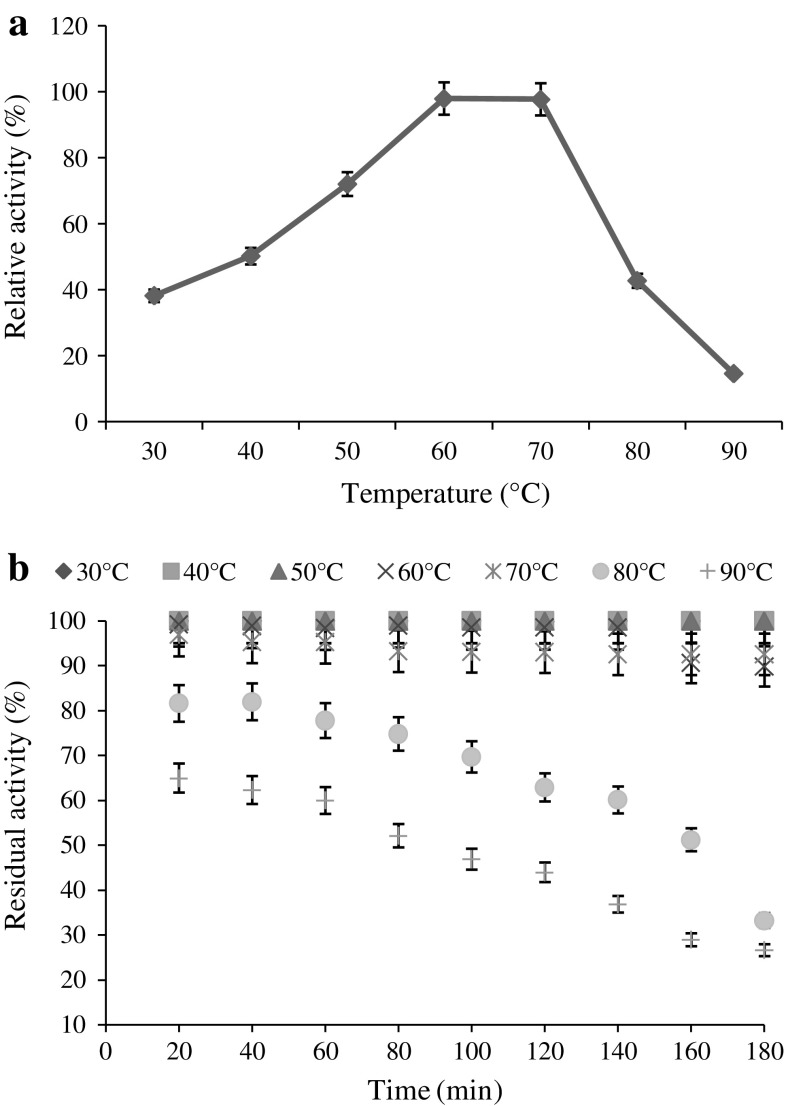
Effect temperature on enzyme activity (**a**) and stability (**b**)

The alpha-amylase produced by *B. amyloliquefaciens* KCP2 was assessed at different pH and temperature ranges and it was observed that the activity was very low at acidic pH, but increase in the pH led to gradual increase in enzyme activity. The maximum activity of the enzyme was observed at pH 8.0 and was found to be highly stable in alkaline pH (Fig. 2a, b). The optimum temperature for alpha-amylase activity was found to be 65 °C and it showed good stability in the temperature range of 30–90 °C. More than 85 % of residual activity was observed in case of temperature ranging from 30 to 70 °C (Fig. 3a, b).

### Screening of significant parameter for alpha-amylase production using Placket–Burman design

A statistical approach has been used to screen the most effective supplement and select their concentration to achieve highest possible alpha-amylase production by *B. amyloliquefaciens* KCP2 under solid-state fermentation using wheat bran as solid substrate. Usually, initial screening of the ingredients is done to understand the significance of their effect on the product formation and then a few better ingredients are selected for further optimization. Plackett–Burman design was used to screen eight different medium components as carbon and nitrogen sources as a 12-run experiment with two levels of concentration of each variable. Studies were carried out under solid-state fermentation at 37 °C for 72 h. The medium components selected as independent variables and their respective high and low concentrations used in optimization study are presented in Table 1, whereas the Plackett–Burman experimental design followed for the optimization of medium components for alpha-amylase production for 12 trials with two levels of concentration of each variable is given in Table 2. The variable X1–X8 represented the medium constituents and D1–D3 represented the dummy variable/unassigned variables. The result of Plackett–Burman experiment with respect to amylase production, the effect, standard error, *t*(xi), *p* and confidence level of each component are represented in Tables 2 and 5. The components were screened at the confidence level of 95 % on the basis of their effects. When components show significance at or above 95 % confidence level and its effect is negative, it is considered effective for production but the amount required may be lower than the indicated low (−1) concentration in Plackett–Burman experiment. If the effect is found positive, a higher concentration that the indicated high value (+) concentration is required. In our experiment, starch, ammonium sulphate and calcium chloride gave confidence level >95 % and could be considered significant and were short-listed for further optimization of their required concentration and their interaction effect leading to maximum enzyme production. Remaining components such as lactose, yeast extract, soya bean meal, ammonium nitrate and dextrose showed confidence level <95 % and were considered insignificant in the study. The methodology of Plackett–Burman was thus found to be very useful for determination of relevant variables for further optimization.

**Table 5:** Statistical analysis of components for alpha-amylase production by *B. amyloliquefaciens* KCP2

Components	Effect	Standard error	*t* value	*P*	Confidence (%)
Dextrose	−3.18	1.64	−1.98	0.140	85.91
Starch	−10.06	1.64	−6.29	0.008	99.18
Lactose	−4.90	1.64	−3.06	0.054	94.53
NH_4_NO_3_	−1.93	1.64	−1.21	0.312	68.77
Yeast extract	0.238	1.64	0.148	0.891	10.88
NH_4_SO_4_	−12.71	1.64	−7.94	0.004	99.58
CaCl_2_	−11.40	1.64	−7.12	0.005	99.43
Soy bean meal	−1.20	1.64	−0.75	0.505	49.47

### Response surface methodology

The Central Composite design was employed to study the interaction among the significant components and also determine their optimal levels. In the present work, experiments were planned to obtain a quadratic model consisting of 2^3^ trials. The plan includes 20 experiments and two levels of concentration for each component. In order to study the combined effect of these medium components, experiments were performed at different combinations. Table 4 summarizes the Central Composite experimental plan along with the predicted and observed response for each individual experiment. It shows the production of alpha-amylase (U/gds) corresponding to combined effect of all three components in the specified ranges.

The optimum levels of the selected variables were obtained by solving the regression equation and by analysing the response surface contour and surface plots (Abdelhay et al. 2008). The regression equation obtained after the analysis of variance (ANOVA) provides an estimate of the level of alpha-amylase production as a function of starch, ammonium sulphate, and calcium chloride concentration.

The production of alpha-amylase may be best predicted by the following model:

5 $$\begin{gathered} {\text{Alpha-amylase production }}\left( Y \right)\,{ = }\,\left( { 4 7. 9 4} \right){-}\left( { 3. 0 8 { } \times \, A} \right)\;{ + }\;\left( { 1. 1 1 { } \times \, B} \right)\;{ + }\;\left( {{ 3} . 5 1 { } \times \, C} \right) - \left( { 1. 0 0 { } \times \, A \, \times \, B} \right) \, \hfill \\ {-}\left( { 0. 6 1 \times \, A \, \times \, C} \right)\;{ + }\;\left( { 2. 2 2 \times \, B \, \times \, C} \right)\;{ + }\;\left( { 0. 0 9 { } \times \, A^{2} } \right) \;{ + }\;\left( { 0. 1 9 \times \, B^{2} } \right)\;{ + }\;\left( { 1. 6 8 { } \times \, C^{2} } \right), \hfill \\ \end{gathered}$$

where *Y* is alpha-amylase production (U/gds), *A* is starch concentration (w/w), *B* is ammonium sulphate concentration (w/w), and *C* is calcium chloride concentration (mM).

The statistical significance of the second-order model equation was evaluated by *F* test analysis of variance which revealed that this regression is statistically highly significant for alpha-amylase production. The model *F* value of 17.98 implies that the model is significant. There is only a 0.01 % chance that a large “Model *F* value” could occur due to noise. Values of “Prob > *F*” <0.05 indicate that the model terms are significant. In this case *A*, *B*, *C*, *BC* and *C* ^2^ are significant model terms (Table 6). The “lack of fit *F* value” of 3.23 implies the lack of fit is not significant relative to the pure error. Non-significant lack of fit is good for the model to fit. The *R* ^2^ value (multiple correlation coefficient) closer to 1 denotes better correlation between observed and predicted values. The coefficient of variation (CV) indicates the degree of precision with which the experiments are compared. The lower reliability of the experiment is usually indicated by high value of CV. In the present case, a low CV (3.22 %) denotes that the experiments performed are reliable. Adequate precision measures the signal-to-noise ratio. A ratio >4 is desirable. In our case, the ratio is of 17.51, which indicates an adequate signal. This model can be used to navigate the design space.

**Table 6:** Analysis of variance (ANOVA) for response surface quadratic model of alpha-amylase production from *B. amyloliquefaciens* KCP2

Source	Sum of squares	*df*	Mean square	*F* value	*p* value	
Model	407.6871	9	45.29857	17.97587	<0.0001	Significant
A-starch	130.1606	1	130.1606	51.65175	<0.0001	Significant
B-Ammonium sulphate	17.12545	1	17.12545	6.795908	0.0262	Significant
C-Calcium chloride	168.6796	1	168.6796	66.93727	<0.0001	Significant
AB	8.142854	1	8.142854	3.231337	0.1025	
AC	3.058689	1	3.058689	1.213782	0.2964	
BC	39.48642	1	39.48642	15.66943	0.0027	Significant
A^2^	0.12979	1	0.12979	0.051505	0.8250	
B^2^	0.54180	1	0.5418	0.215003	0.6528	
C^2^	40.94109	1	40.94109	16.24669	0.0024	Significant
Residual	25.19965	10	2.519965			
Lack of fit	19.24826	5	3.849652	3.23425	0.1118	Not significant
Pure error	5.951384	5	1.190277			
Core total	432.8868	19				

CV 3.22 %, adequate precision 17.51

**Fig. 4:**
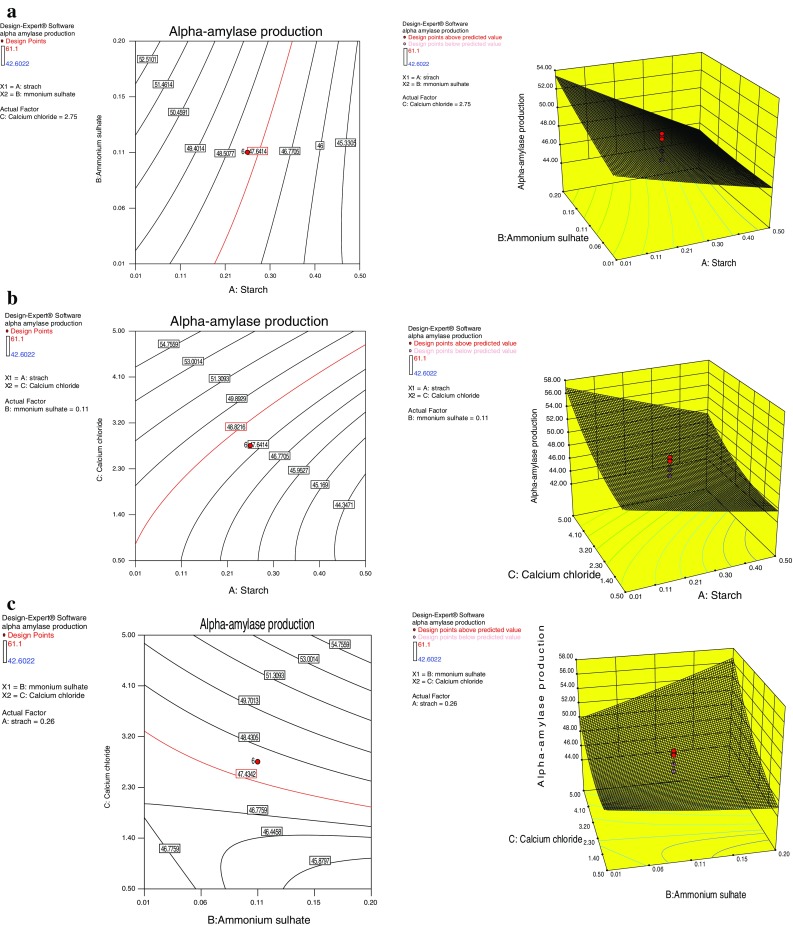
Response surface graph showing interaction effects between concentration of starch and ammonium sulphate (**a**), starch and calcium chloride (**b**), and ammonium sulphate and calcium chloride (**c**)

The effect of interaction of variables on enzyme (alpha-amylase production) yield was studied against any two independent variables while keeping the other independent variables at their constant level. These response surface plots or contour plots can be used to predict the optimal values for different test variables. Therefore, three response surfaces were obtained by considering all the possible combinations. Three-dimensional response plot shown in Fig. 4a describes the behaviour of alpha-amylase production, main effect, interaction effect and squared effect (nonlinear) of starch and ammonium sulphate at different concentrations. Both the components at their lower level did not show any significant effect on the alpha-amylase production. The shape of the response surface curves showed a moderate interaction between these tested variables. Increase in the starch concentration leads to gradual decrease in the enzyme production, while increase in the ammonium sulphate concentration results in the significant increase in the alpha-amylase production. Figure 4b depicts three-dimensional curve and contour plot of the calculated response surface from the interaction between starch and calcium chloride while keeping fixed concentration of ammonium sulphate. The level of the calcium chloride in the production medium showed prominent effect on the alpha-amylase production. Increase in the calcium chloride concentration leads to concomitant increment in the alpha-amylase yield, while both components at their lower level did not result in the higher enzyme yield. Figure 4c depicts the interaction of ammonium sulphate and calcium chloride where the shape of the response surface indicates positive interaction between these two factors. The enzyme yield was found to increase with simultaneous increase in concentration of both the components. Both the components at their lower level did not show any significant effect on the enzyme yield.

### Validation of the quadratic model

Validation was carried out under conditions predicted by the response surface model. The optimal concentrations estimated for each variable were 0.01 g starch, 0.2 g ammonium nitrate and 5 mM calcium chloride per 5 g of wheat bran. The predicted alpha-amylase production obtained from the model using the above optimum concentration of medium components was 61.48 U/gds. To validate the prediction of the model, additional experiments in triplicate were performed with the optimized medium. These experiments yielded the maximum amylase activity of 63.12 U/gds. Good agreements between the predicted and experimental results verified the validity of the model and the existence of the optimal points.

## Discussion

Amylases have several interesting potential application in the food, detergent, pharmaceutical, leather, textile, cosmetic, and paper industries. Still their application in starch-based industries is the major market and thus demand of amylases would always be very high in this sector. There is a constant search for microorganisms producing enzyme with desired properties of pH and temperature stability considering their industrial application. In this respect the isolate KCP2 was found to have production of enzyme with such properties and was characterized further. As shown in the Fig. 1, the evolutionary history was inferred using the neighbor-joining method (Saitou and Nei 1987). The optimal tree with the sum of branch length of 1.51712318 is shown and the percentage of replicate trees in which the associated taxa clustered together in the bootstrap test (500 replicates) are shown next to the branches (Felsenstein 1985). The evolutionary distances were computed using the maximum composite likelihood method (Tamura et al. 2004) and are in the units of the number of base substitutions per site. Codon positions included were 1st + 2nd + 3rd + non-coding and all positions containing alignment gaps and missing data were eliminated only in pairwise sequence comparisons (pairwise deletion option). There were a total of 1,579 positions in the final dataset.

A marked enhancement in production of alpha-amylase by *B. amyloliquefaciens* in flask fermentation using statistical methods was reported by Zhao et al. (2011). Use of Plackett–Burman design to screen different nutrients affecting production of thermostable β-amylase and pullulanase by *Clostridium thermosulfurogenes* SV2 has been reported by Reddy et al. (1999). The substrate employed in the present investigation, i.e. wheat bran, has been reported as a potent substrate for production of alpha-amylase under SSF (Gangadharan et al. 2006; Ramachandran et al. 2004; Mulimani et al. 2000). It is well documented that wheat bran is rich source of carbon and nitrogen, thus supplementation of other nitrogen sources in the medium does not show any significant rise in the enzyme production but sometimes presence of starch as an additional carbon source was found to have inductive effect, and it also has remarkable efficiency in the production of enzyme, being an inexhaustible source of carbon compared to other carbon sources (Prajapati et al. 2013). In the present investigation, apart from starch, lactose was also found to act as an inducer for enzyme production as it gave 94.53 % confidence level during components screening study. Added nitrogen sources also have been reported for an inducing effect for the production of various enzymes including alpha-amylase in an SSF system (Pedersen and Nielsen 2000; Akher et al. 1973). Earlier reports show that among various inorganic nitrogen sources tested, ammonium sulphate, ammonium chloride and ammonium hydrogen phosphate favour growth and enzyme secretion (Narang and Satyanarayana 2001).

Alpha-amylase is an inducible enzyme, which is generally induced in the presence of starch or its hydrolytic product maltose (Rama and Srivastav 1995). *Bacillus thermooleovorans* preferred starch, glucose, lactose, maltose and maltodextrin as favourable carbon sources for amylase secretion (Narang and Satyanarayana 2001). In some cases, hydrolyzed starch and glucose were found to repress the enzyme yield, which may be due to feedback inhibition caused by the presence of reducing sugars. Easily metabolizable carbohydrates may result in the better growth of the organism along with reduction in the enzyme formation (Rama and Srivastav 1995). The supplementation of metal ions has been reported to provide good growth and also influence higher enzyme yield (Sivaramakrishnan 2006). Most of the alpha-amylase is metalloenzymes and in most of the cases, Ca^+2^ ions are required for maintaining the spatial conformation of the enzyme, thus play an important role in the enzyme stability and its activity. Amylase from the halophilic *Bacillus* sp. Strain *TSCVKK* showed stability at a wide pH range of 6.5–10.5 with maximum activity with pH 8.0 suggesting the alkalitolerant nature (Kondepudi and Chandra 2008). Majority of the literature showed that 50 °C is the optimum temperature for alpha-amylase activity, but in our case we found that 65 °C is the optimum temperature for the activity of enzyme produced by *B. amyloliquefaciens* KCP2. Temperature and pH stability of this enzyme justify its alklophilic and thermophilic nature. Statistical approach has also been applied for the production of various enzymes, such as cyclodextrin glucanotransferase (CGTase) (Gawande and Patkar 1999; Mahat et al. 2004), chitinase (Gohel et al. 2005), pectinase (Nair and Panda 1997), vitamin riboflavin (Punjari and Chandra 2000) and glucoamylase (Prajapati et al. 2013).

Present investigation has allowed rapid screening and level optimization of a large number of nutrient parameters influencing thermophilic and alklophilic alpha-amylase production from *B. amyloliquefaciens* KCP2 using statistical methodology. The results also showed the use of cheap agro-residue as substrate for fermentation, thus contributing to the reduction in cost of production medium. The enzyme yield and the production were found to be significantly influenced by starch, ammonium sulphate and calcium chloride concentration. The data obtained after optimization has resulted in 63.12 U/gds enzyme production. Even though SSF is widely applied for enzyme production using filamentous fungi, the results of the present study proved that a bacterial isolate such as *B. amyloliquefaciens* KCP2 can be successfully used for the production of alpha-amylase employing wheat bran within a relatively shorter time interval of 3 days. 
